# The impact of a 12-week high-fructose diet on the metabolic syndrome and ischaemia-reperfusion injury in rats

**DOI:** 10.1038/s41598-026-73467-x

**Published:** 2026-10-03

**Authors:** David C. Mathew, Siavash Beikoghli-Kalkhoran, Alan D. Salama, Sean M. Davidson, Derek M. Yellon

**Affiliations:** 1 The Hatter Cardiovascular Institute, University College London, 67 Chenies Mews, London, WC1E 6HX UK; 2 Royal Free Hospital, Faculty of Medical Sciences, University College London Medical School, University College London, London, UK

**Keywords:** Fructose, Cardio-metabolic, Ischemia-reperfusion, Rodent model, Uricase, Cardiology, Diseases, Physiology

## Abstract

Excess fructose consumption is implicated globally in metabolic pathologies, ultimately resulting in cardiac mortality. As such, robust animal models of fructose-induced pathology, with a cardiac phenotype, are required to investigate underlying disease processes. We evaluated the impact of a 12-week high-fructose diet on cardiometabolic parameters and susceptibility to ischaemia–reperfusion injury in Wistar rats. Our protocol demonstrated increased fructose intake and systemic exposure, accompanied by enlargement of the liver and renal hypertrophy. Despite this, functional cardiac impairment was not demonstrated via echocardiographic assessment of systolic or diastolic function. In addition, increased dietary fructose exposure was not associated with increased susceptibility to ischaemia-reperfusion injury in an ex vivo Langendorff model of global ischaemia, and no gross inflammatory or fibrotic changes were evident on histological examination. Explanations for the lack of observed cardiac phenotype may include the necessity of additional physiological stressors e.g. high fat or salt intake as well as the presence of an active uricase enzyme in rodents. These considerations could explain the heterogeneity of outcomes observed in available animal models and the disparity with human observational data, where the negative impacts impact of increased dietary fructose intake are readily observed.

**Supplementary Information:**

The online version contains supplementary material available at https://doi.org/10.1038/s41598-026-73467-x.

## Introduction

Fructose is a monosaccharide sugar prevalent in the Western diet, consumption of which is associated with a diverse range of metabolic pathologies, including chronic kidney disease (CKD) and cardiac morbidity. This deleterious effect can be attributed to the rate limiting enzyme in the “fructolytic” pathway, ketohexokinase (KHK) activity of which ultimately results in uric acid generation, inflammation and suppression of oxidative phosphorylation^1^.

Clinical evidence for the adverse effect of excessive fructose consumption includes interventional studies in human subjects demonstrating increased visceral adiposity in response to fructose ingestion as well as impaired glucose tolerance and significant dose dependent increases in LDL cholesterol, triglycerides and uric acid^2,3^. Meanwhile, observational data demonstrate a significant association between fructose intake and non-alcoholic fatty liver disease (NAFLD), albuminuria and CKD^4–6^. Finally, increased fructose intake is significantly associated at a population level with development of hypertension, cardiovascular morbidity and coronary heart disease^7,8^. As such these multi-system sequelae clearly place fructose consumption and metabolism as a central mediator in the “cardiovascular-kidney-metabolic syndrome” (CKMS), a disorder attributable to connections among obesity, diabetes, kidney disease and cardiovascular disease, the common endpoint being cardiovascular mortality^9,10^.

Investigation of the biological processes driving the adverse consequences of excessive fructose intake relies on a variety of approaches, including robust animal models of disease. Aspects of fructose-induced pathology have been observed in a variety of rodent models, including insulin resistance, hepatic steatosis, hypertension and CKD^11–14^. Given that cardiac mortality represents the common end pathway in CKMS, it is essential to investigate the effects of fructose intake on cardiac outcomes and develop models of fructose-induced cardiac injury. Previous work has demonstrated diastolic dysfunction in rodent models of high fructose intake, consistent with human disease^15^. Furthermore, in the chronic setting, high fructose diet in rodent models of cardiac ischemia-reperfusion (IR) injury has been observed to increase infarct size and impair indices of cardiac function e.g. Rate Pressure Product (RPP) and E/A ratio^16–21^. However, while individual studies have identified specific outcomes associated with excess fructose consumption, there is a still a marked heterogeneity in the literature with regards to model design, leading to inconsistent phenotypes. Variables include species (i.e. mouse or rat), strain of animal, mode of fructose delivery (solid food vs. dissolved in drinking water), fructose content of feed (e.g. 10%), additional composition of feed (e.g. high fructose + high fat) and duration of study. Given that cardiac mortality represents the “end point” of fructose associated harm, we sought to develop a fructose feeding protocol which would induce features of the “metabolic syndrome” as well as fructose-induced cardiac dysfunction, such that the underlying pathological mechanisms could be studied in further detail. A well-characterised outcome in this context is susceptibility to myocardial “ischemia-reperfusion injury”, the paradoxical effect by which restoration of blood flow results in increased cardiomyocyte death^22^.

Based on available literature, we chose to design our protocol using fructose as the only addition to the intervention diet (as opposed to fructose + high fat, for example). This was to avoid the confounding effects of high fat or high salt intake on cardiac outcomes. Additionally, we chose to add fructose to the solid food pellet rather than to the drinking water. This was to enable accurate matching of energy intake between control and intervention groups. Mouse studies have demonstrated greater energy intake when sucrose is offered in water than when offered the same macronutrient composition but in solid form^23^. The increased energy intake associated with “water-sucrose” resulted in greater adiposity, possibly due to a lack of satiety when carbohydrate calories are consumed in liquid rather than solid form^23^.

To develop a protocol capable of inducing *cardiac* injury in particular, we reviewed previous animal studies utilising high fructose diet in solid feed which investigated cardiac infarct size following ischaemia-reperfusion injury. In addition, we only considered studies of greater than six weeks duration - this is because acute fructose administration in the context of ischaemia has been shown to be cardioprotective, which could be related to acute rapid ATP production via anaerobic glycolysis, the inflammatory sequelae of which are detrimental in the chronic setting^24^. We identified two studies fitting these inclusion criteria, both of which utilised Wistar rats for a period of either 12 or 24 weeks with fructose concentrations of 30% and 35%, respectively^17,18^. Based on these studies, we chose to use Wistar rats for the present study and a time course of 12 weeks. To maximise dietary fructose exposure, we chose to use a diet containing 60% fructose calorie matched to a control diet of normal chow. This diet has been well tolerated in studies using Sprague Dawley, Wistar rats and C57B1/6J mice for periods of 4–15 weeks^21,25,26^. A recent systematic review has also identified that increasing concentrations of fructose in pellet diets reproduce more of the associated parameters of fructose-induced metabolic syndrome (e.g. hypertension) and recommend these for use in future studies, such as our own^11^.

**Hypothesis**: A 60% fructose diet administered for 12 weeks to Wistar rats will induce features of the cardio-metabolic syndrome and increase cardiac infarct size when subjected to ischemia-reperfusion injury.

## Results

### A 60% fructose diet is well tolerated and results in increased systemic fructose exposure

**Fig. 1:**
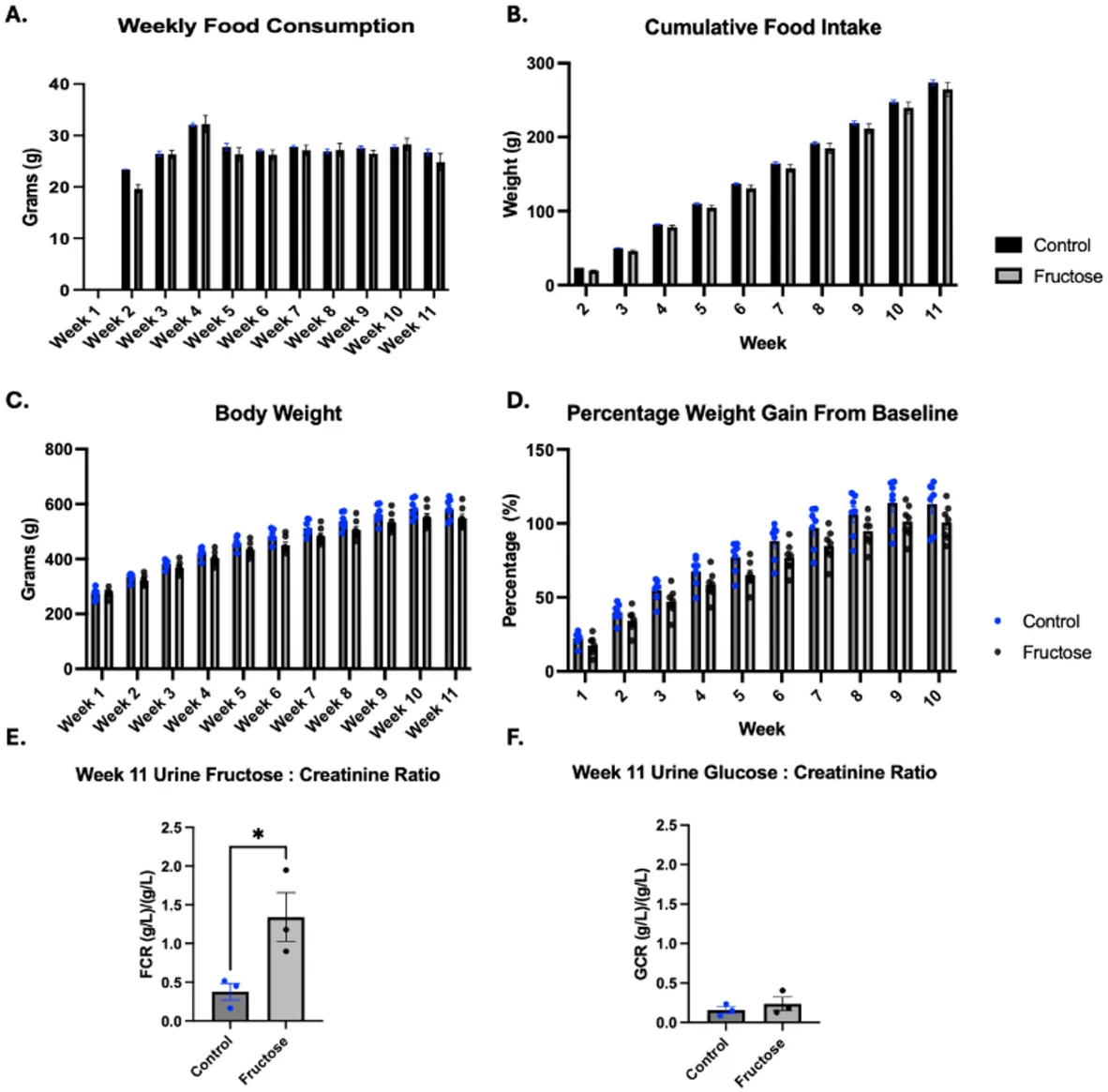
Fructose Exposure. **(A)** Weekly food consumption. Data are reported per cage (*n* = 3) as animals were not singly housed. There was no significant effect of diet on food consumption, *p* = 0.42. **(B)** Cumulative food intake. There was no significant effect of diet on cumulative food intake: (*n* = 3) *p* = 0.30. **(C)** Body weight. There was no significant effect of diet on body weight, (*n* = 3) *p* = 0.10. **(D)** Percentage weight gain from baseline. There was no significant effect of diet on percentage weight gain (*n* = 7) *p* = 0.10 **(E)** Week 11 urine fructose: creatinine ratio (uFCR), there was a significant increase in uFCR in fructose fed animals, (control 0.38 ± 0.10 vs. fructose 1.34 ± 0.31) *p* = 0.044, *n* = 3. **(F)** Week 11 urine glucose: creatinine ratio (uGCR). There was no difference in uGCR between groups (control 0.16 ± 0.04 vs. fructose 0.24 ± 0.08) *p* = 0.41, *n* = 3. **A**, **B**,** C**,** D** analysis by two-way ANOVA with Geisser Greenhouse correction and Tukey post-test. **C** and **D** analysis by unpaired students t-test, *= *p* < 0.05.

Food consumption and body weight of both study groups were measured weekly (Fig. 1). There was no significant effect of diet on food consumption, either by weekly measurement or by cumulative intake. Of note, the diet consumption in both groups was lower than the subsequent average values in study week two, possibly due to an acclimatisation effect. This was then compensated for in weeks two to four before reaching steady levels from week five onwards. There was no significant effect of diet on body weight between groups, either by weekly measurement or percentage change from baseline, indicating that the diet was tolerable to the intervention group and both groups consumed diet equally. To ensure diet consumption resulted in increased systemic fructose exposure, we measured urine fructose-to-creatinine ratio (*n* = 3 rats per group) at week 11. A significant increase was observed in the fructose-fed group indicating a higher systemic fructose exposure. There was no difference in the glucose-to-creatinine ratio between groups.

### Increased fructose intake is associated with hepatomegaly and renal hypertrophy but does not reconstitute other features of the “Metabolic Syndrome”

**Fig. 2:**
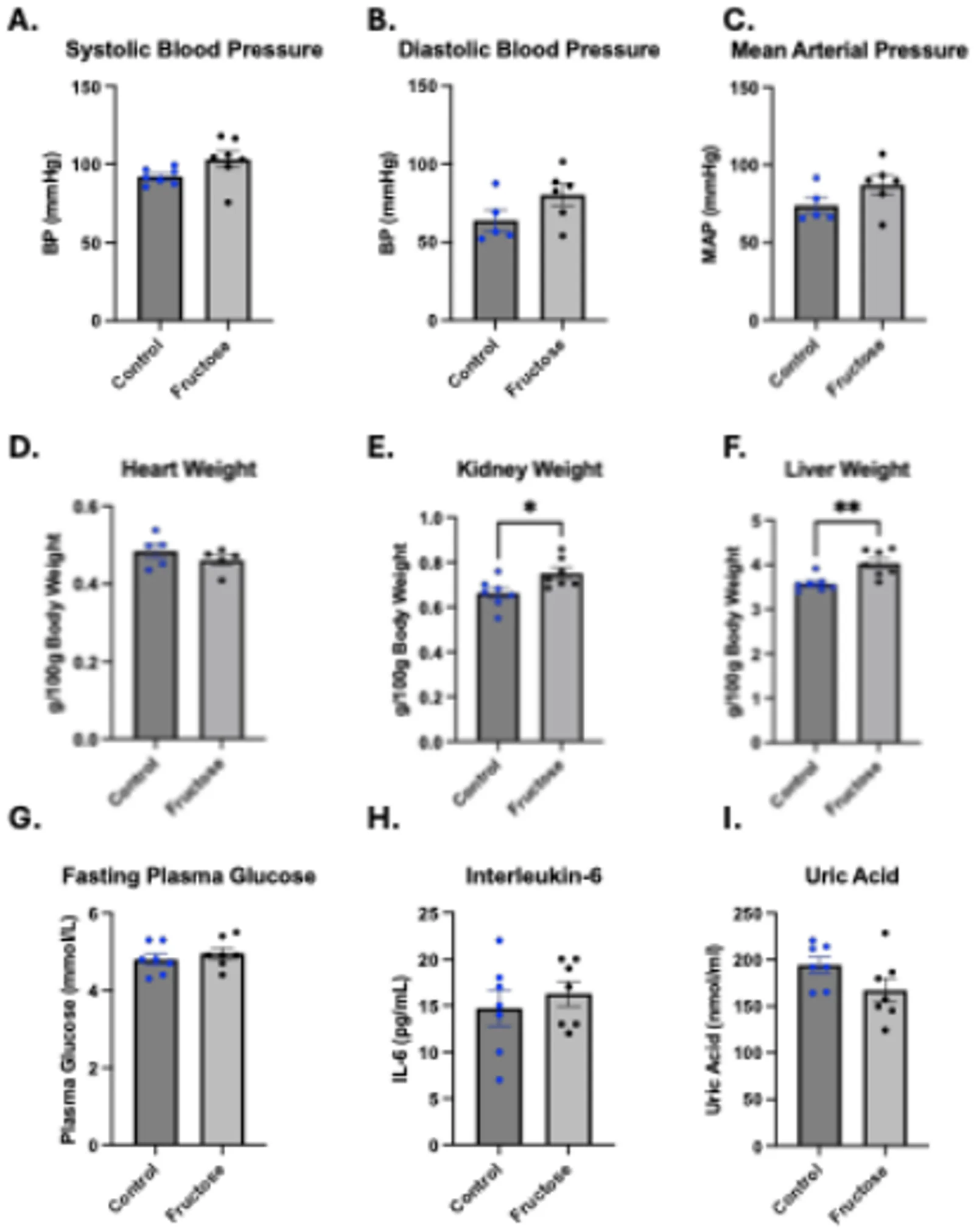
Blood pressure, organ weight and plasma markers. **A + B.** There was no significant difference in end of study systolic or diastolic blood pressure between groups. **(A)** Systolic: (control: 92.1 ± 1.9 vs. fructose 103.1 ± 5.3) *n* = 7, *p* = 0.07. **(B)** Diastolic: (control 63.9 ± 6.6 fructose 80.3 ± 6.8) *n* = 7, *p* = 0.12. **(C)** There was no significant difference in mean arterial pressure between groups (control 73.8 ± 5.0 Vs fructose 87.1 ± 6.2) control *n* = 5, fructose *n* = 6, *p* = 0.13. **(D)** There was no significant difference in heart weight adjusted for body weight, (control 0.48 ± 0.02 vs. fructose 0.46 ± 0.01) *n* = 5, *p* = 0.34. **(E)** There was a significant increase in kidney weight when adjusted for body weight in the fructose fed group (control: 0.66 ± 0.002 vs. fructose: 0.75 ± 0.002) *n* = 7, *p* = 0.026. **(F)** There was a significant difference in liver weight adjusted for body weight (control 3.57 ± 0.1 vs. fructose 4.04 ± 0.1) *n* = 7, *p* = 0.004. **(G)** There was no significant difference in end of study fasting blood glucose measurement: (control: 4.80 ± 0.15 vs. fructose: 4.96 ± 0.14) *n* = 7, *p* = 0.46. **(H)** There was no significant difference in end of study plasma IL-6: (control: 194.2 ± 8.7 vs. fructose 167.2 ± 12.9) *n* = 7, *p* = 0.1. **(I)** There was no significant difference in end of study plasma uric acid concentration: (control: 14.71 ± 0.9 vs. fructose 16.29 ± 1.3) *n* = 7, *p* = 0.51. Analysis by unpaired students t-test, *= *p* < 0.05, **=*p* < 0.005.

To further characterise the phenotype induced by this protocol, we investigated several additional physiological parameters which are commonly disturbed in models of high fructose intake. We observed a trend towards hypertension in systolic and diastolic blood pressure measurements of fructose fed animals, however this did not attain significance (Fig. 2.). Additionally, there was no significant difference in mean arterial pressure (MAP). There was also no effect of high fructose diet on heart weight.

Consistent with previous fructose-feeding studies characterised by renal hypertrophy and glomerular hyperfiltration, we identified increased kidney weight normalised to body weight (Fig. 2)^14^. Consistent with the known effects of high-fructose consumption in development of NAFLD we identified a significant increase in liver weight when adjusted for body weight in the fructose fed group (Fig. 2). There was no significant difference in fasting plasma glucose at the end of our study. With regard to plasma markers, there was no significant difference in plasma IL-6 or uric acid at the end of the study.

### High fructose diet is associated with hepatic lipid deposition

**Fig. 3:**
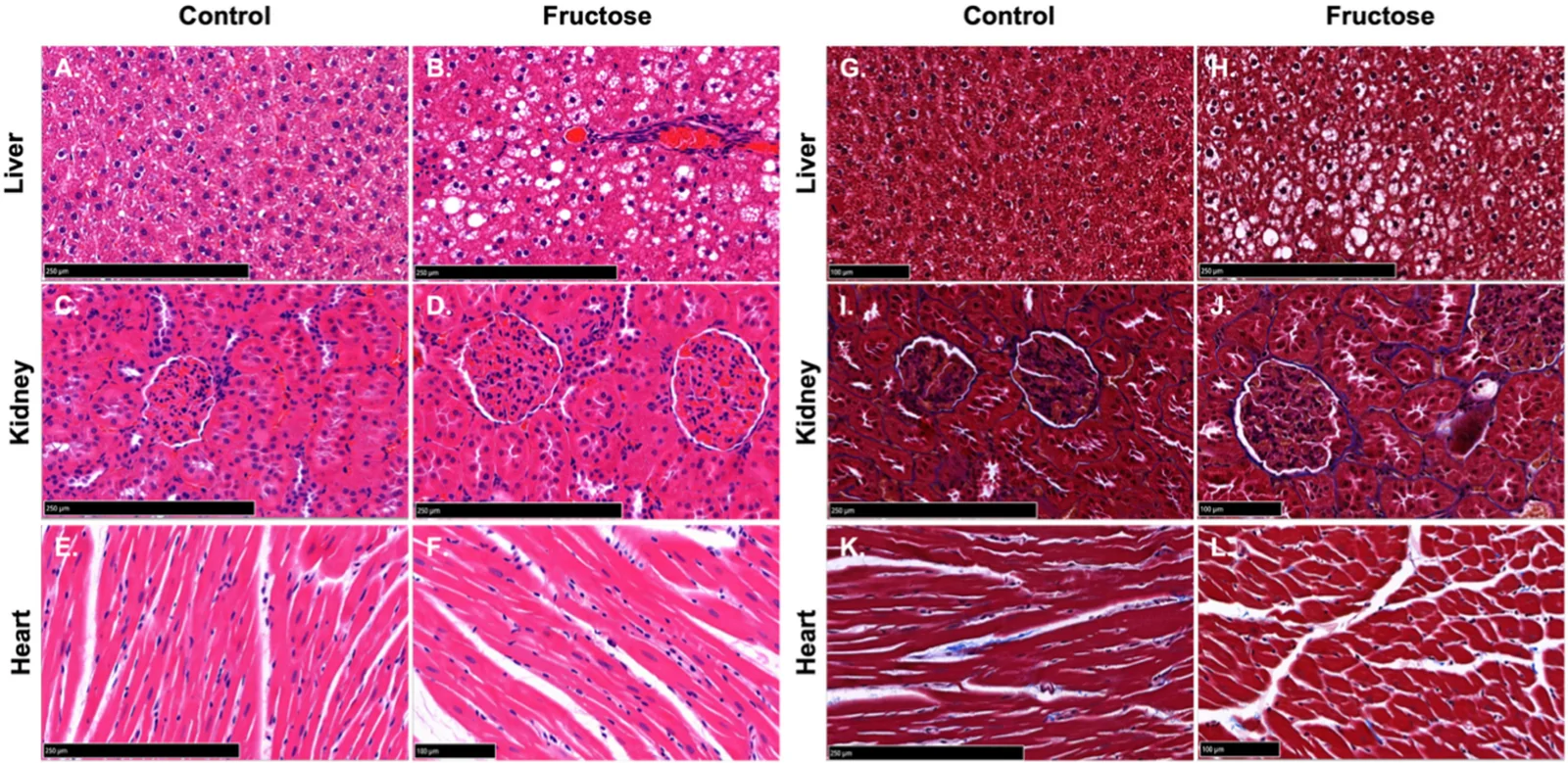
Immunohistochemical analysis of liver, kidney and heart tissue. **(A-F)** Haematoxylin and eosin-stained sections from liver, kidney and heart tissue of control and fructose-fed animals. Lipid droplet deposition is observed in the parenchyma of fructose fed animals with no increase in inflammatory cell infiltrate. There are no gross morphological changes observed in kidney or heart tissue between groups. The same organs were stained with Masson’s trichome **(G-L)**, no obvious increase in fibrosis was noted in liver, kidney or heart in the fructose fed group. Images presented at 20x magnification.

We further examined sections of liver, kidney and heart by immunohistochemistry. In keeping with increased liver length and weight observed, the liver of fructose-fed rats demonstrated the presence of lipid deposition. There were no gross morphological differences in kidney and heart and no obvious inflammatory cell infiltrate observed on hematoxylin and eosin stain (H&E) or fibrosis observed on Massons’ trichome (Fig. 3).

### High-fructose diet induces upregulation of renal ketohexokinase

**Fig. 4:**
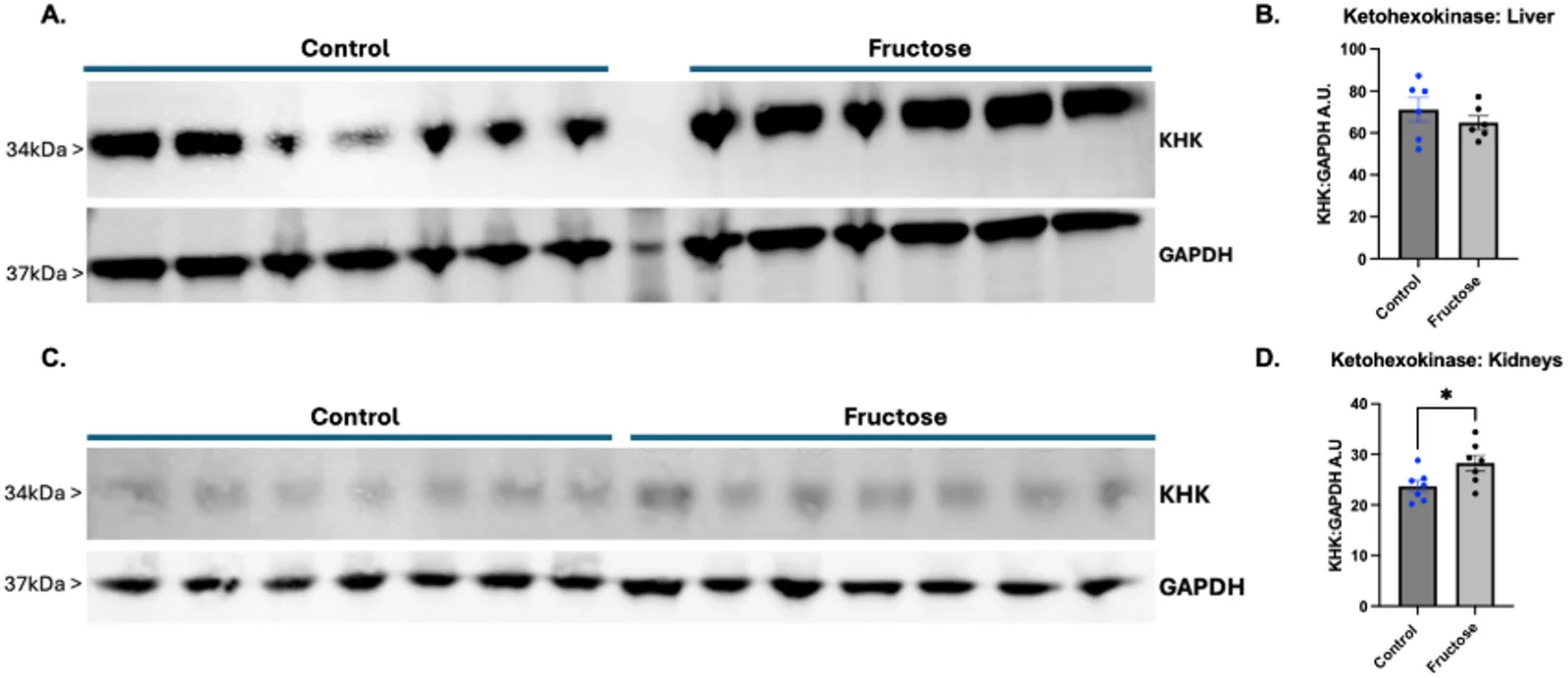
Hepatic and renal ketohexokinase protein quantitation. **(A)** Western blot analysis of livers extracted from control and fructose-fed animals at the end of the study protocol. Tissue lysate was probed for ketohexokinase (KHK) and GAPDH. Control *n* = 7, Fructose *n* = 6. **(B)** Quantitation of protein from the blot pictured in (A). There is no significant increase between quantity of KHK protein detected in livers of fructose fed rats, (control: 71.3 ± 5.7 vs. fructose 65.0 ± 3.2), *p* = 0.37. **C.**Western blot analysis of kidneys extracted from control and fructose fed animals at the end of the study protocol. Tissue lysate was probed for ketohexokinase (KHK) and GAPDH. *N* = 7. **D.** Quantitation of protein from the blot pictured in **(C).** There is a significant increase in KHK protein detected in kidneys of fructose fed rats. (control: 23.7 ± 1.1 vs. fructose 28.3 ± 1.5), *p* = 0.034. Results are expressed in Arbitrary Units (A.U.) Analysis by unpaired students t-test, *= *p* < 0.05. Original blot is available in supplementary Fig. 1.

As an additional indicator of fructose exposure, we measured ketohexokinase protein in liver and kidney. KHK is the rate limiting enzyme in the fructolytic pathway and is upregulated in response to fructose exposure, in addition, activity of KHK is associated with fructose-induced renal damage^27^. In our study we found no significant increase of KHK in the liver of fructose fed animals (Fig. 4). However we did find a significant increase in renal KHK in the fructose fed rats.

### Increased fructose exposure does not affect echocardiographic indices of cardiac function

**Fig. 5:**
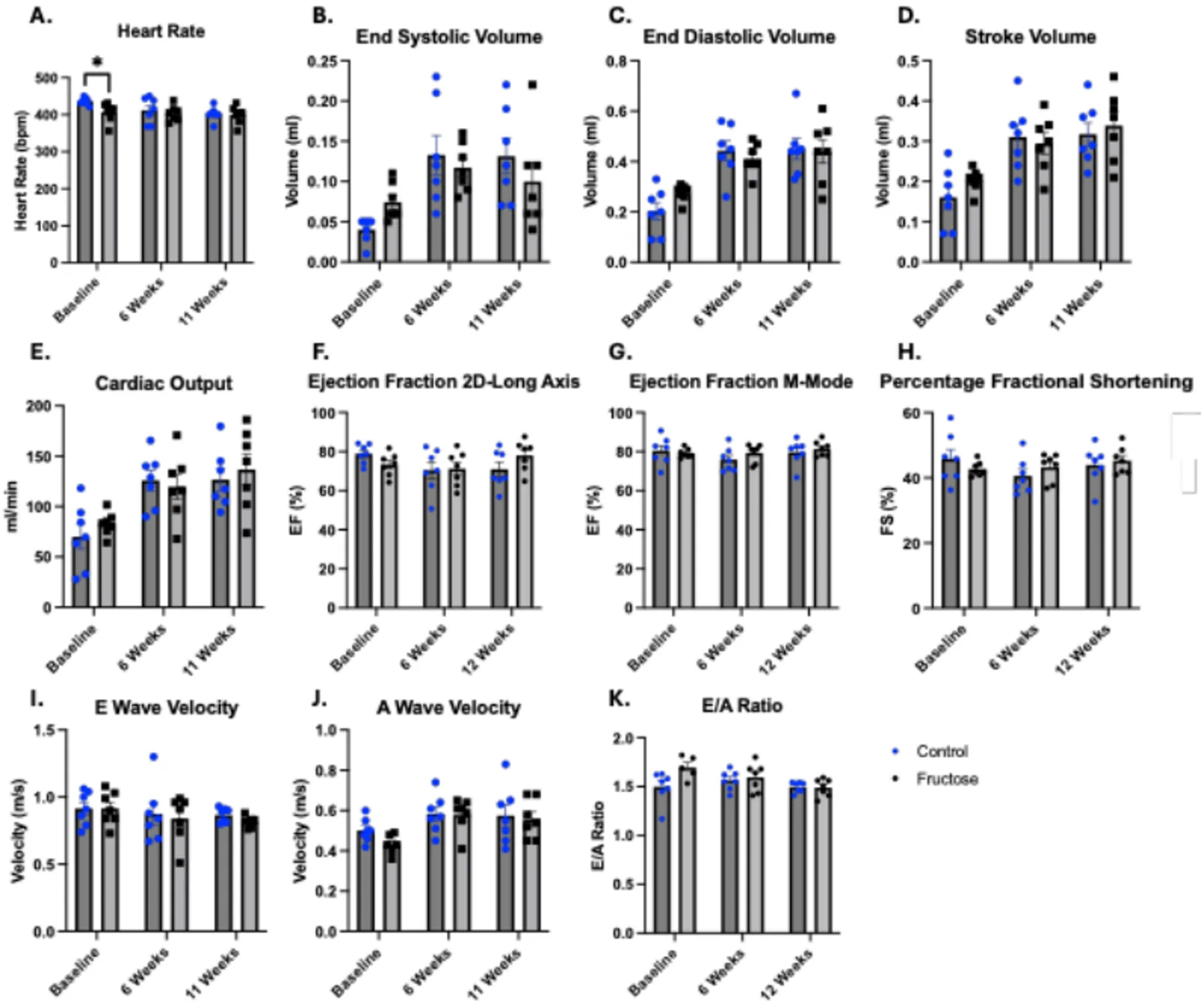
Echocardiographic assessment. **A.** Heart rate. There was no significant effect of diet on heart rate (*p* = 0.1) A mildly reduced heart rate was detected in the fructose group at the study outset by Tukey post-test (*p* = 0.02).**B**,** C**,** D.** There was no significant effect of diet on end systolic volume, end diastolic volume or stroke volume (*p* = 0.78, *p* = 0.77, *p* = 0.6 respectively). **E.** There was no significant effect of diet on cardiac output (*p* = 0.6). **F.** Ejection fraction (EF) measurement via 2D long axis. There was no significant effect of diet on EF, (*p* = 0.78). **G.** EF via M-mode. There was no significant effect of diet on EF, (*p* = 0.51). **H**. Percentage fractional shortening. There was no significant effect of diet on percentage fractional shortening, (*p* = 0.24). **I**,** J** There was no significant effect of diet on E or A wave velocity (*p* = 0.55 and *p* = 0.34 respectively) **K.** E/A Ratio. There was no significant effect of diet on E/A ratio, *p* = 0.052. (*n* = 7) A-H analysis by Two-Way ANOVA with Geisser Greenhouse correction and Tukey post-test, I, J, K Analysis by mixed effects model with Geisser Greenhouse correction and Tukey post-test.

To assess the effect of increased fructose exposure on cardiac function we performed echocardiography on the rats to measure indices of systolic and diastolic function at baseline, midpoint and at the end of the study. We did not find any significant effect of high-fructose diet on heart rate or indices of systolic function (See Fig. 5A-H). In addition, we were not able to detect a significant effect of fructose intake on indices of diastolic function including measurements of E and A wave velocity as well as E/A ratio - an echocardiographic index of left ventricular diastolic performance reflecting ventricular filling dynamics (Fig. 5).

### Increased fructose exposure was not associated with increased susceptibility to ischaemia-reperfusion injury

**Fig. 6:**
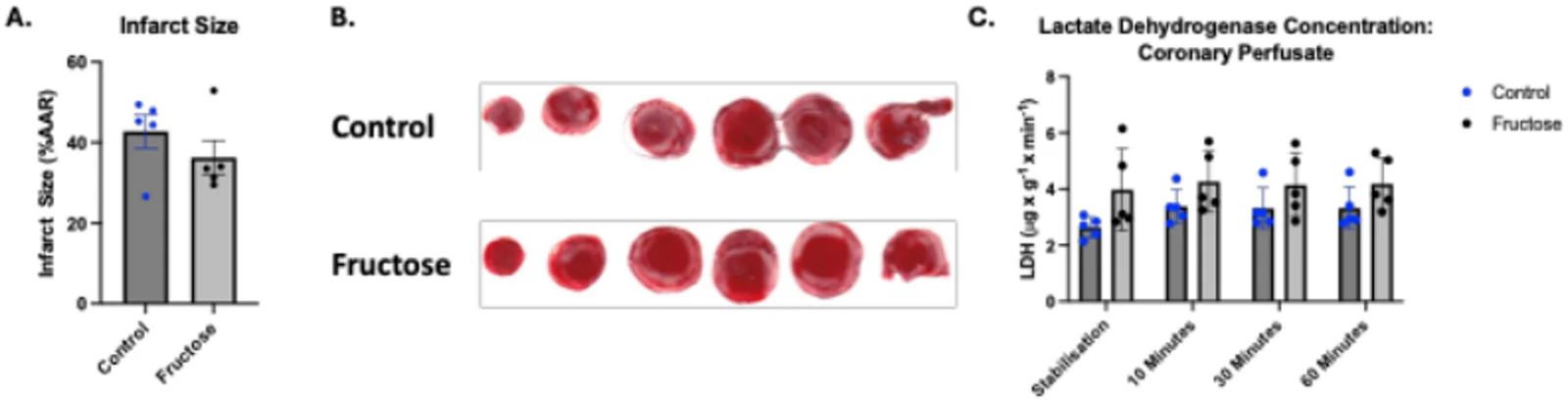
Primary outcomes: myocardial infarct size and coronary perfusate lactate dehydrogenase concentration after ischemia-reperfusion injury. **(A)** Infarct Size measured as a percentage of area at risk (%AAR). There was no significant difference between the control and fructose treated groups: (control 42.72 ± 4.12 vs. fructose 36.25 ± 4.23) *p* = 0.31, *n* = 5. **(B)** Representative images of heart slices stained with triphenyl tetrazolium chloride used for infarct planimetry analysis. **(C)** Coronary perfusate lactate dehydrogenase concentration. There was no significant effect of diet on perfusate LDH concentration: *p* = 0.12. A analysis by unpaired students t-test, C analysis by two-way ANOVA with Geisser Greenhouse correction and Tukey post-test.

The primary outcome measure from our study was susceptibility to ischaemia-reperfusion injury measured by myocardial infarct size and coronary perfusate lactate dehydrogenase (LDH) concentration (an enzyme associated with cell death and myocardial injury) in an ex vivo model of myocardial ischaemia-reperfusion, in which the hearts were subject to 35 min global ischaemia followed by 120 min reperfusion (*n* = 5 hearts per group). There was no difference in infarct size between control and fructose groups (Fig. 6). Additionally, there was no effect of diet on perfusate LDH concentration during reperfusion.

## Discussion

In the present study, a fructose-feeding protocol was developed based on extensive literature review with the aim of inducing metabolic disturbance and fructose-induced cardiac dysfunction. Notably, the literature base is characterised by heterogeneity, both in study design and outcomes, despite the readily observed detrimental effects of increased dietary fructose intake in human observational studies and it’s central role in the “cardiovascular-kidney metabolic syndrome”^7,11,16–18,21,28^. Given that the final common endpoint in CKMS is increased cardiac mortality, our protocol was designed with the intention of inducing a detrimental cardiac phenotype, such that underlying pathological processes can be investigated^9^.

Principally we found that, although some features of the metabolic syndrome were evident e.g. liver and kidney enlargement, accompanied by hepatic lipid droplet deposition, we were unable induce cardiac dysfunction in the form of increased susceptibility to ischemia-reperfusion injury and functional assessment via echocardiogram. This is despite the increased systemic fructose exposure evident in our model in the form of increased urine fructose concentration, and the upregulation of renal ketohexokinase.

Previous work demonstrating increased susceptibility to ischaemia-reperfusion (I/R) injury in response to a high-fructose diet has used Wistar rats for a minimum duration of 12 weeks^17,18,29^. The current study is consistent with this duration, however differs from these models by using a higher concentration of fructose (60%). This compares with, 30% fructose in solid feed, 10% fructose in drinking water or a combination of 35% in solid feed and 25% in drinking water, all of which have increased infarct size after I/R injury^17,18,29^. The decision to use a 60% fructose diet, which has previously been well tolerated by Wistar rats^25^ was based on a desire to maximise dietary fructose exposure, as increasing concentrations of fructose in pellet diets reproduce more of the associated parameters of fructose-induced metabolic syndrome^11^. A 60%-fructose diet has also previously induced cardiac dysfunction in Sprague Dawley rats with success, albeit after 20 weeks of treatment^21^. Our data demonstrate adequate diet consumption compared with control and increased systemic fructose exposure evidenced by increased urine fructose concentration and upregulation of renal ketohexokinase. Together, these considerations suggest that systemic fructose exposure appears to have been adequate in the context of a 60%-fructose diet, which has previously been associated with cardiac injury.

A possible explanation for the lack of observable cardiac phenotype in this study is the duration, which was limited to 12 weeks of 60% fructose intake. There is precedent for a duration of 24 weeks of high fructose intake producing increased infarct size after I/R injury; however increased infarct size has been demonstrated by others in Wistar rats after only 12 weeks of high fructose intake^17,18^. With regard to cardiac function, previous work has demonstrated a reduction in both ejection fraction and E/A ratio in Sprague Dawley rats fed 60% fructose for 12 weeks^21^. Overall 12 weeks appears to be an adequate duration for a study of this nature.

Another consideration relating to the lack of observable cardiac phenotype is the complexity of the Western diet consumed in the “real world”. The benefit of supplementing animal diet with fructose alone in basic science models is the removal of confounding factors e.g. “high fat” or “high salt” on outcomes. However, it is possible that high fructose intake merely “sets the scene” for the development of a full “cardio-metabolic syndrome” in the presence of these additional stressors. This phenomenon has been observed in rodent studies, where high fructose intake predisposes to salt-sensitive hypertension as demonstrated by Garvin et al.^13,30^. It is possible therefore that development of a reliable cardio-metabolic phenotype requires a “second hit” on a background of “fructose conditioning”, this could be dietary e.g. “high salt”, or strain related e.g. use of Zucker Fatty or Spontaneously Hypertensive rats.

The potential requirement for multiple dietary stressors is highlighted by the lack of increased diet intake or body weight observed in this study. High fructose consumption is associated with obesity in human studies^2^ and is known to suppress leptin. Our study is consistent with previous work however in not demonstrating increased food intake or body weight. Individual studies have reported no differences in body weight or calorie intake in response to 60%-fructose diet for a period of 24 weeks despite demonstrable leptin suppression^30^. Furthermore, a meta-analysis of solid-feed high-fructose diet models demonstrates no significant effect on body weight or calorie intake^28^.

A further consideration regarding the phenotype observed in this model may be related to the lack of raised plasma uric acid in the high fructose diet group. Increased fructose consumption directly elevates plasma uric acid in humans and is the origin of much of fructose-induced harm. A key difference between rodents and humans is that rodents express an active uricase enzyme, which affords protection from the deleterious consequences of fructose metabolism and provides resistance to the development a “cardio-metabolic” phenotype. Confirmation of this is provided by exacerbation of fructose-induced renal injury with inhibition of uricase^31^. This observation may also explain the lack of raised inflammatory markers in the form of IL-6 in our study and provides an alternative option for future work: namely a combination of high-fructose intake and a uricase inhibitor.

Our study is limited in several respects. While qualitative immunohistochemical analysis demonstrates fatty liver infiltration and a lack of morphological changes in the kidney, this has not been quantitatively assessed. While we did not demonstrate hypertension in our rodents, these measures were taken non-invasively and under isoflurane anaesthesia. It is possible that invasive blood pressure monitoring may have demonstrated hypertension. We also acknowledge that these experiments were undertaken in male rats only, the reason for this being the demonstrable protective effect of female sex hormones in the development of fructose-induced metabolic syndrome, and our intent to maximise the model phenotype for further study^32^. Finally, we measured fasting blood glucose at the end of our study however additional measures e.g. fasting insulin and HOMA-IR index could have provided more information regarding glucose tolerance.

In conclusion, we aimed to induce metabolic disturbance and cardiac injury via the development of a protocol of high dietary fructose intake in Wistar rats. While we were able to observe some metabolic features, our primary outcome was neutral. Possible explanations for this include inadequate duration of study and the confounding effects an active uricase enzyme in rodents. We also suggest that this data, combined with the heterogenous literature base hints at the action of fructose as a “conditioning agent” rather than an end effector. High fructose exposure may pre-dispose an organism to the development of a cardio-metabolic syndrome which requires a “second hit” e.g. high salt intake. Framing the problem in this way may aid development of further animal models. This is necessary as understanding the underlying mechanisms governing fructose-induced cardiac dysfunction is increasingly pertinent in the Western world.

## Methods

### Animal study design

**Fig. 7:**
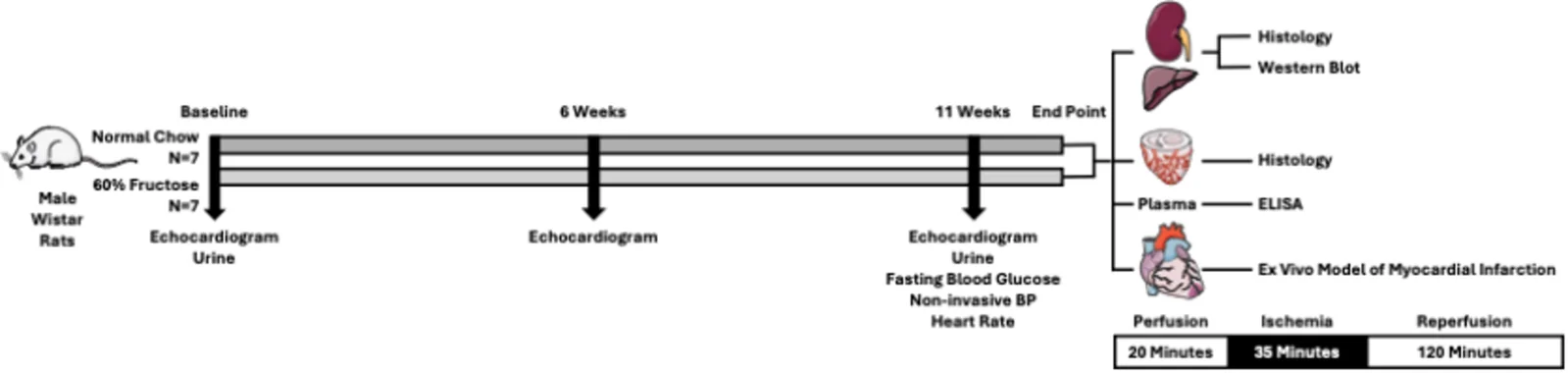
Study protocol. High Fructose Diet (HFD) vs. Control Study protocol summary.

All experiments were carried out in accordance with the Home Office (United Kingdom) Guidance on Research and Testing using animals and the Animals (Scientific Procedures) Act of 1986. The procedures performed in this study were part of the project license PP9987686, approved by the animal welfare and ethical review board of UCL and the Home Office. All experiments complied with ARRIVE guidelines. Male Wistar rats (*N* = 14) aged 8 weeks old were obtained from a single supplier (Charles River UK), housed under identical conditions and randomly allocated to receive control diet of 60%-fructose diet for a period of 12 weeks (*N* = 7 per group) (Fig. 7).

The composition of both diets is listed in Table 1; both were obtained from a single supplier (Ssniff) and matched for calorie content. Diet consumption and animal weight were measured weekly throughout the study period. As animals were not housed singly, diet consumption per rat was calculated weekly by normalising the gross amount of diet consumed to the number of animals in the cage (e.g. two or three animals). As there were three cages per experimental condition, these values are presented as *n* = 3 per group. Echocardiography was undertaken at baseline, week 6 and week 11. Urine fructose: creatinine ratio (uFCR) and urine glucose: creatinine ratio (uGCR) were measured at baseline and 11 weeks. Additional measurements at 11 weeks included: fasting plasma glucose and non-invasive blood pressure and heart rate monitoring. The study period concluded at 12 weeks whereupon hearts (*n* = 5 per group) were subject to ex vivo ischaemia-reperfusion injury. Organs and plasma were also collected for further analysis. (Fig. 7)

**Table 1:** Diet composition.

Control diet	Fructose diet
Crude Protein 17.6%	Crude Protein 17.6%
Crude Fat 7.1%	Crude Fat 7.1%
Crude Fibre 4.0%	Crude Fibre 4.0%
Crude Ash 3.2%	Crude Ash 3.2%
Starch 51.2%	Starch 3.8%
Sugar 11.2%	Sugar 61%
Vitamin A 4000 IU/kg	Vitamin A 4000 IU/kg
Vitamin D3 1000 IU/kg	Vitamin D3 1000 IU/kg
Vitamin E 75 mg/kg	Vitamin E 75 mg/kg
Copper 10 mg/kg	Copper 10 mg/kg

### Echocardiography, blood pressure measurement, heart rate measurement

Echocardiogram was undertaken using a “Vivid I” machine (GE Healthcare). Animals were anaesthetised via inhaled isoflurane (2 L/min) and body temperature maintained using a heating mat. Measurements were taken of: heart rate, end systolic volume, end diastolic volume, stroke volume, cardiac output, ejection fraction by 2-D long axis, ejection fraction by M-mode, percentage fractional shortening, E wave velocity, A wave velocity, and E/A ratio. The values presented relating to diastolic function are an average of three individual measurements. Blood pressure measurements were measured non-invasively via tail cuff inflation using a “Non-invasive blood pressure system for rodents” (Harvard Apparatus). Values presented are an average of two readings.

### Urine fructose and glucose : creatinine ratios

Urine D-Glucose, D-Fructose and creatinine were measured using commercially available absorbance assays and used to calculate uFCR and uGCR, (Neogen, USA) (Abcam, UK).

### End of study period tissue and plasma collection

At the end of the study period animals underwent terminal anaesthesia using intraperitoneal pentobarbitone (200 mg/kg) and concurrently anticoagulated with heparin 400–650 IU/kg consistent with previous studies^33,34^. Upon confirmation of deep anaesthesia whole blood was collected by cardiac puncture and the plasma separated by centrifugation at 2000 RCF for 15 min at 4 °C. Supernatant was stored at -80 °C. Tissue samples e.g. liver (*n* = 7 per group), kidneys (*n* = 7 per group), tibia (*n* = 7 per group) and hearts (*n* = 2 per group) were collected, weighed and measured before snap freezing in liquid nitrogen or preservation in 4% paraformaldehyde.

### Ex Vivo Langendorff model of global ischaemia-reperfusion injury

As above, animals underwent terminal anaesthesia at the end of the study period. Upon confirmation of deep anaesthesia hearts (*n* = 5 per group) were surgically excised and mounted on a 16-gauge cannula using 2x silk sutures. For the duration of the subsequent experiment hearts were perfused at a constant pressure of 80 mmHg with warmed modified Krebs-Henseleit buffer composed of: NaCl 118 mM, NaHCO3 25 mM, d-Glucose11 mM, KCL 4.7 mM, MgSO4.7H20, 1.22 mM, KH2PO4 1.21 mM, CaCl2.2H20, 1.84 mM. The buffer was aerated with a mixture of O2 (95%) and CO2 (5%) to maintain a pH of 7.42 +/- 0.3^35^. Temperature was maintained at 37 °C ± 0.5 °C and a fluid filled balloon was inserted into the left ventricle attached to a pressure transducer and inflated to targe Left Ventricular End Diastolic Pressure (LVEDP) < 20mmHg.

Hearts were perfused for an initial 20-minute stabilisation period and excluded from analysis of any of the following criteria were met: Time to cannulation > 4 min, LVEdP > 20 mmHg, developed pressure < 70 mmHg, coronary flow rate < 10 ml/min or > 28 ml/min, heart rate < 70 bpm or > 400 bpm. (85)

After the stabilisation period, hearts were subject to global ischaemia for a period of 35 min. This was followed by a reperfusion period of 120 min. Coronary effluent samples were collected at 10, 30 and 60 min post reperfusion and stored at -20 °C for later analysis of LDH concentration. At the end of the reperfusion period hearts were removed from the Langendorff apparatus, dried and weighed before being stained with 2,3,4 tetrazolium triphenylchloride (TTC) to assess infarct size^36^.

### TTC staining and analysis of infarct size

Hearts were cut into 2 mm sections along the short axis and incubated in a 1% TTC solution for 20 min at 37 °C and transferred to 10% neutral formalin buffer for at least 1 h before imaging with a Canon LiDE 220 scanner at 1200 dpi resolution.

Infarct size planimetry was performed using ImageJ (Version 1.53). Infarct size was calculated as a percentage of left ventricle (LV) and adjusted for mass.

### Lactate dehydrogenase concentration in coronary perfusate samples

Lactate dehydrogenase concentration was measured via colourimetry using the CytoTox 96^®^ Non-Radioactive Cytotoxicity Assay, (Promega, UK) and absorbance measured by microplate reader (BMG Labtech 96) at 492 nm. A standard curve was generated using known concentrations of recombinant human LDH (Abcam, UK) and used to calculate absolute LDH concentration (µg/ml). This value was adjusted for heart mass and coronary flow to produce an expression of LDH concentration (µg x g^− 1^ x min^− 1^) This is consistent with previous methods ^37^. LDH is an enzyme released from damaged cardiomyocytes and is an established marker of myocardial injury^36^.

### Western blot analyses

Kidney and liver tissue lysates were obtained from samples homogenised in RIPA lysis buffer composed of: Tris 100mM (ThermoFisher), NaCl 300mM, IGEPAL (NP40) 1% (Sigma), made up to 100 ml with double distilled H20, pH adjusted to 7.4. Homogenisation was carried out using a Potter Elvehjem homogeniser and Vibracell Sonicator before centrifugation at 10,000 rpm for 10 min at 4 °C. Protein concentration was calculated using BCA assay (Pierce 23223) and 30 ug of protein per sample separated by SDS-PAGE (10% NuPAGE NP0301BOX) before transfer to 0.45 μm PVDF membrane (Millipore). Blocking buffer comprising of 1 x Tris Buffered Saline supplemented with 0.1% TWEEN20 (TBST) and 5% powdered skimmed milk (Cell Signalling Technology) was applied for 1 h before incubation with mouse or rabbit primary antibodies: Ketohexokinase: (sc-377411, 1:100)^38^ and GAPDH (sc-25778, 1:1000). Membranes were washed with TBST for 3 × 10 min prior to staining with secondary antibodies: IRDye^®^ 680LT Goat Anti-Mouse (LI-COR) and IRDye^®^ 800CW Goat Anti-Rabbit (LI-COR) diluted 1:15000 and 1:20000 respectively in TBST supplemented with 5% powdered milk. Membranes were incubated with secondary antibodies for 1 h before washing for 10minx2 with TBST and imaged using LI-COR Odessey, version 3.0. Protein quantitation values were normalised for GAPDH and expressed as arbitrary units (A.U.).

### Immunohistochemistry

Kidneys (*n* = 7 per group) and liver sections (*n* = 7 per group) and hearts (*n* = 2 per group) fixed in 4% paraformaldehyde overnight and were subsequently placed in phosphate buffered saline (PBS). Sections were stained with haematoxylin and eosin (H&E) and Masson’s trichome staining.

### Plasma biomarkers

Plasma Interleukin 1 (IL-6) and Uric Acid were analysed using commercially available sandwich assays: (R&D Systems R6000B), (AbCam ab65344).

### Statistics

Data were analysed using Graphpad Prism V 10.6.1. Unpaired Student’s t-test, or one-way ANOVA with Tukey post-test were applied where appropriate. Where repeated measures of grouped data are presented, a two-Way ANOVA or mixed effects model Geisser Greenhouse correction with Tukey post-test were used. Data are presented as mean ± SEM.

### Power calculation

Calculation of statistical power was made using the nomographic method of Altman with statistical significance 5% and power 85%. Based on literature review and preliminary studies in our laboratory we estimated an effect size of 20% in our primary outcome, namely infarct size after ischaemia-reperfusion injury.(135, 139) As such a study size of 7 animals per group was selected.

## Supplementary Information

Below is the link to the electronic supplementary material.

Supplementary Material 1

## Data Availability

All data available on reasonable request.
