# Supplementary material for: The impact of a 12-week high-fructose diet on the metabolic syndrome and ischaemia-reperfusion injury in rats
Source: Sci Rep. 2026 Oct 3;16:33823. doi: 10.1038/s41598-026-73467-x (PMC13694245; doi:10.1038/s41598-026-73467-x)

**Challenges and Considerations in the Development of a Rodent Model of Fructose Induced Cardio-Metabolic Syndrome. Supplementary Data**

Supplementary Figure 1: Original Western Blots of Liver and Kidney Ketohexokinase Protein

Figure 1 A: Liver Western Blot

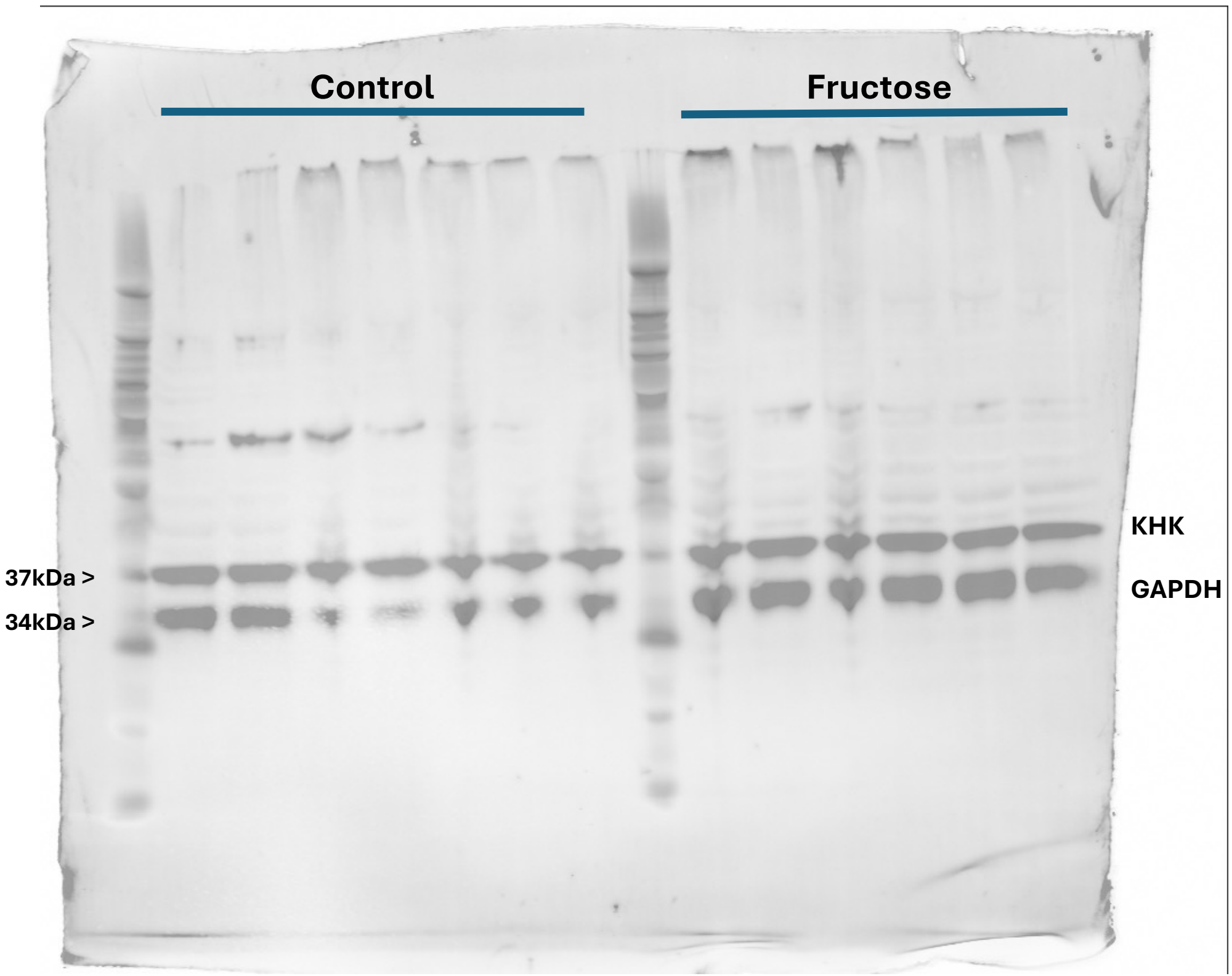

Figure 1B: Kidney Western Blot

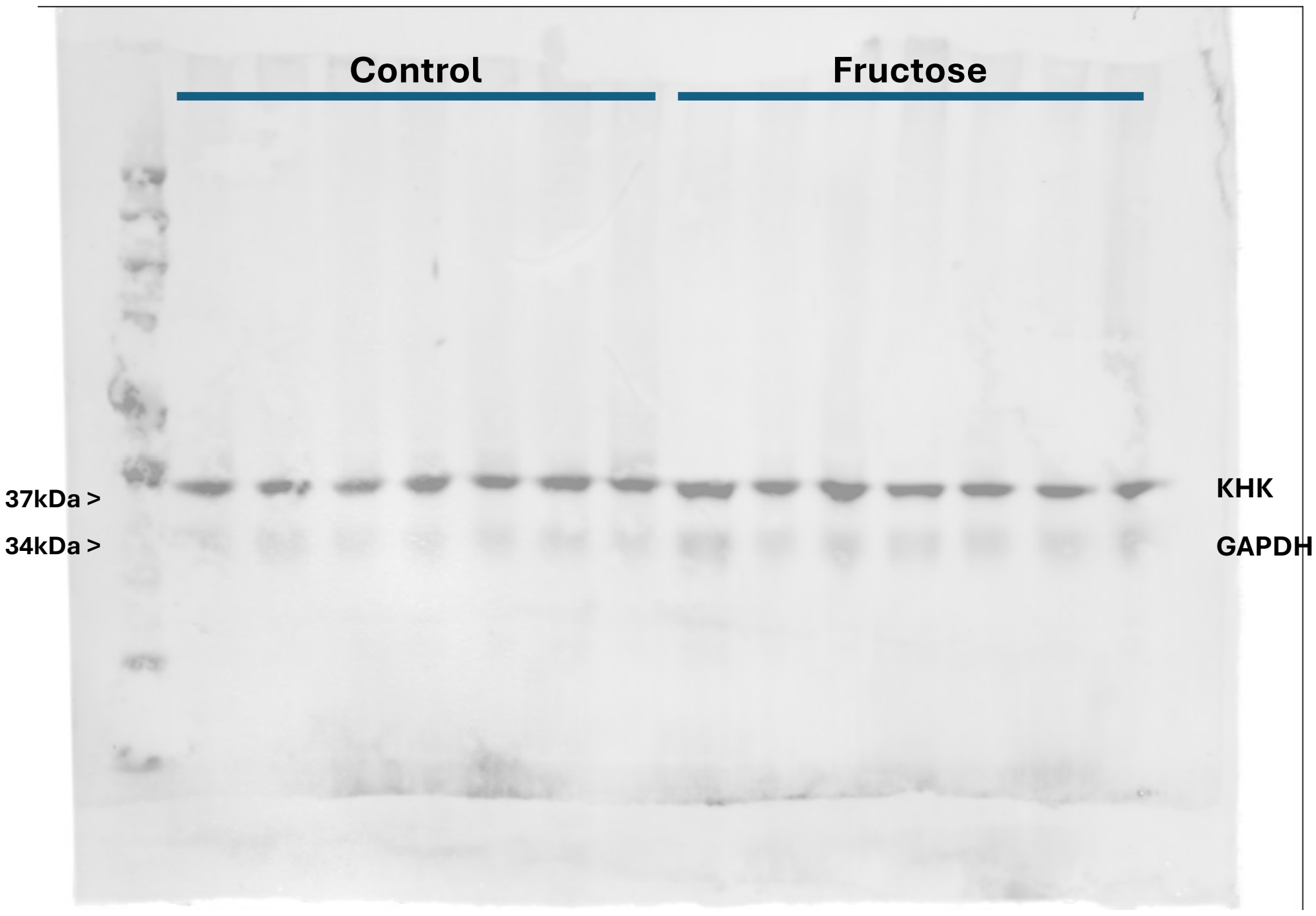

Supplement: Supplementary file 1 [file 41598_2026_73467_MOESM1_ESM.pdf]
